# A 3D Laser Profiling System for Rail Surface Defect Detection

**DOI:** 10.3390/s17081791

**Published:** 2017-08-04

**Authors:** Zhimin Xiong, Qingquan Li, Qingzhou Mao, Qin Zou

**Affiliations:** 1 State Key Laboratory of Information Engineering in Surveying, Mapping and Remote Sensing, Wuhan University, Wuhan 430079, China; sagittarius@whu.edu.cn (Z.X.); qzhmao@whu.edu.cn (Q.M.); 2 Shenzhen Key Laboratory of Spatial Smart Sensing and Services, Shenzhen University, Shenzhen 518060, China; 3 School of Computer Science, Wuhan University, Wuhan 430079, China; qzou@whu.edu.cn

**Keywords:** rail surface defect, defect detection, iterative closest point, laser imaging

## Abstract

Rail surface defects such as the abrasion, scratch and peeling often cause damages to the train wheels and rail bearings. An efficient and accurate detection of rail defects is of vital importance for the safety of railway transportation. In the past few decades, automatic rail defect detection has been studied; however, most developed methods use optic-imaging techniques to collect the rail surface data and are still suffering from a high false recognition rate. In this paper, a novel 3D laser profiling system (3D-LPS) is proposed, which integrates a laser scanner, odometer, inertial measurement unit (IMU) and global position system (GPS) to capture the rail surface profile data. For automatic defect detection, first, the deviation between the measured profile and a standard rail model profile is computed for each laser-imaging profile, and the points with large deviations are marked as candidate defect points. Specifically, an adaptive iterative closest point (AICP) algorithm is proposed to register the point sets of the measured profile with the standard rail model profile, and the registration precision is improved to the sub-millimeter level. Second, all of the measured profiles are combined together to form the rail surface through a high-precision positioning process with the IMU, odometer and GPS data. Third, the candidate defect points are merged into candidate defect regions using the K-means clustering. At last, the candidate defect regions are classified by a decision tree classifier. Experimental results demonstrate the effectiveness of the proposed laser-profiling system in rail surface defect detection and classification.

## 1. Introduction

The rail surface defect is an important factor that affects the operation safety of rails. Common rail surface defects such as abrasion, corrugation, scratch, corrosion and peeling would cause damage to the train wheels and the rail bearings, which will not only shorten the service life of train parts, but also bring a potential critical safety crisis to the trains. Therefore, the research and development of automatic methods for rail surface defect detection is very important, which on the one hand achieves an effective maintenance of the train-operation safety by timely and accurate defect location and recognition and, on the other hand, improves the passenger comfort and environmental protection, reduces noise and the energy loss caused by rail defects.

Automatic detection of defects from rail surface images is a very challenging problem. In the past two decades, many research works and achievements have been made by using two-dimensional (2D) imaging for rail surface defect detection. However, there are still some problems left unsolved. From the perspective of texture analysis, filtering and wavelet transform-based approaches are widely used for defects detection by analyzing the local spatial patterns, e.g., the Gabor filter, curvelet transform and Gabor wavelet transform [1,2,3,4]. However, due to the heavy computation cost, these methods are incapable of real-time handling of a large number of per-pixel convolution operations on a high resolution rail image. Meanwhile, heavy random noise sometimes may be brought by complicated rail track conditions and camera thermal effects, which make it difficult to discriminate defective regions from the background noise. Another group of research works focuses on intensity histogram analysis for rail surface defect detection, for instance the geometrical analysis method that directly performs a gray-level histogram [5] and the threshold method calculating a threshold at the valley of two peaks or at the left bottom rim of a single peak histogram [6]. Generally, the intensity-based methods heavily depend on the image quality, which would be affected by the defect shape, orientation, surface material, texture, etc. In addition, there may exist many oxide scales on the rail surface, and the color of the oxide scale may be very similar to some surface texture caused by defects such as scarring, indentation, and so on, which makes it difficult to distinguish the defects by intensity analysis. It is worth noticing that real-time automatic detection methods have also been studied. In [7], a real-time visual inspection system was put forward to detect discrete rail surface defects, which was reported to run on a 216-km/h testing speed. In [8], an automatic visual inspection system was developed for detecting partial abrasion by using a probabilistic topic model. However, these systems were tailored to detect some particular rail defects and are limited in the general rail surface detect detection, which requires locating defects of a wide variety and correctly identifying their types.

In this paper, we design and manufacture a three-dimensional laser profiling system (3D-LPS) to achieve high-speed information acquisition on the rail surface under a complex environment. The 3D-LPS integrates the laser scanner, odometer, IMU, GPS, etc. Two laser scanners are used to capture the depth information of the rail surface. Combined with the position of GPS, the attitude of IMU and the mileage of the odometer, the depth data can be used to generate the 3D point cloud of the rail surface. Then, a 3D rail defect detection method is proposed, which first extracts the point cloud of the rail surface and then calculates the deviation between the defective point cloud and the discrete standard rail model. Specifically, an adaptive iterative closet point algorithm is presented to achieve accurate registration of the defective point cloud and the standard model, with a registration accuracy of sub-millimeters. After this, the k-means algorithm is used to cluster the defects and extract the characteristics of each defect area. At last, a decision tree classifier is employed to identify the type of rail surface defect. In practice, five types of common rail surface defects are studied, which are abrasion, corrugation, scratch, corrosion and peeling, and experiments show that the proposed rail surface defect detection method can locate the defects with millimeter precision and classify the defects with a high accuracy rate.

The remainder of this paper is organized as follows. Section 2 overviews the related work on rail surface defect detection. Section 3 introduces the design of the proposed 3D-LPS. Section 4 introduces our rail surface defect detection method. Section 5 reports the experimental results from about a 400-m railway for about 400,000 profiles, and Section 6 concludes the paper. 

## 2. Related Work

### 2.1. Rail Surface Defect Detection

Rail detection technology mainly uses magnetic induction, ultrasonic technology, laser measurement technology and image processing technology. Many research works have been done and many achievements made on this aspect. Toliyat Hamid A. The work in [2] adopts the magnetic coil and Hall sensors to acquire signals and decomposes the signals by applying wavelet coefficients, and the,n a method of detecting rail defects based on the wavelet transform is proposed, which is combined with the autoregressive spectrum, energy monitoring and differentiation dimension. Papaelias M. [9] proposes the alternating current field measurement (ACFM) method by introducing a remote uniform current into an area under test and monitoring the magnetic field variations associated with the current flowing. Clark [10] concludes about the early technologies based on ultrasonic testing. Bartoli I. [11] and Coccia S. [12] of the University of California-San Diego (UCSD) studied the use of ultrasonic waves for long-range rail defect detection and established a guided wave propagation model. The Federal Railway Authority (FRA) and Lanza di Scalea F. [13] of UCSD combine ultrasonic detectors and laser sensors to detect transverse defects under horizontal shelling. Alippi C. [14] and Babenko P. [15] use the laser ranging scanner to obtain the depth of the rail, combined with the matching camera to obtain the optical image, so as to detect rail defects. Deutsch E. [16] takes line-scan cameras and a special image acquisition method of the spectral image differencing procedure to obtain defects on the rail surface and then detects the defects by means of image processing.

In the past few decades, the most common non-destructive testing technique used to detect defects in rails is ultrasonic testing. Rose, Zumpano et al. [17,18] study the guided wave propagation technologies and adopt algorithms like continuous wavelet transform, the ray-tracing algorithm, etc., to extract defects. These technologies perform relatively well in detecting deep surface-breaking and internal defects, but the rail surface defects are usually not detected by this high-speed ultrasonic technique.

In recent years, image recognition technology has been widely used to detect defects based on local spatial patterns of intensity; for instance, the Gabor filter [1] and wavelet transform [2]. Mandriota [3] and Marino [4] compare three filtering approaches (Gabor filter, wavelet transform and Gabor wavelet transform) based on texture analysis of the rail surface to extract texture features for rail corrugation detection. However, these spectral methods are not fit for real-time processing because they involve a large amount of convolution operations per pixel of high resolution rail images. Furthermore, there exists heavy random noise mainly caused by complicated rail track conditions and camera thermal effects, which make it difficult to distinguish noise from the defective region. Xie [19] reviews recent advances in surface inspection using computer vision and image processing techniques, particularly those based on texture analysis methods, and divides the techniques into four categories: statistical approaches, structural approaches, filter-based methods and model-based approaches. Lin [5] adopts geometrical analysis directly on a gray-level histogram curve of the smoothed rail head surface image to locate defects. Li [7] puts forward a real-time visual inspection system (VIS) to detect discrete rail surface defects. VIS acquires rail image and cuts the rail track subimage by the track extraction algorithm, subsequently enhancing the contrast using the local normalization method and, at last, detecting defects using the defect localization based on the projection profile. VIS is very fast and can run on a 216-km/h test train in real time. Feng [8] proposes an automatic visual inspection system for detecting partial abrasion using the probabilistic topic model. Yuan [6] proposes an improved Otsu method named the weight object variance (WOV); the weight ensures that the threshold always is a value that is located at the valley of two peaks or at the left bottom rim of a single peak histogram. However, these image-based technologies heavily depend on image quality, which is directly affected by defect shape, orientation, surface material, texture, etc., and also has some needs for a special light source, which is difficult to integrate and is expensive. Furthermore, there are many oxide scales on the rail surface, and the color of the oxide scale is very similar to some surface defects, such as scarring, indentation, and so on; therefore, it is difficult to distinguish by the use of a two-dimensional image. More importantly, these technologies can only recognize particular rail defects and cannot classify several defects correctly. With the rapid development of laser sensors, the acquisition of fast and high-precision point cloud data becomes possible and applicable on surface information, which solves the difficulty of information acquisition on the rail surface under a complex environment. In the past few years, the 3D vision system, especially the line-structured optical scanning system, has been applied to many aspects of surface defect detection [20,21,22,23,24], but there have been few mature automatic defect extraction algorithms proposed.

### 2.2. Defect Point Registration

3D point cloud data registration is the most critical part of surface defect detection. In order to compare with the standard data, it is necessary to register the collected point cloud data with the discrete standard CAD model to facilitate the corresponding coordinate difference. The registration algorithm is widely used in machine vision and image processing. The main processes include: feature extraction, feature representation, matching calculation and evaluation. According to the different features, the 3D point cloud registration algorithm can be divided into internal features matching and external features matching. The internal features are independent of the coordinate system, such as the curvature and the distance between two points, and the external features are related to the coordinate system, for instance the tangent plane of the surface [25]. The internal features matching is a reduced dimension and indirect matching method; it includes the surface signature method [26,27], the spin-image method [28], the geometric histogram method [29], the harmonic shape image method [30], the splashes method [31], etc. Because of the reduced dimension processing, the calculation speed is faster, but the precision is not high. The most commonly-used algorithm in external features matching is the label method [32,33] and the optimal matching algorithm. The label method requires pasting at least three markers or positioning balls on the measured object as a physical feature point. Through the coordinates of the three points, the position between the coordinate system can be calculated. In order to improve the accuracy of the calculation, more labels or positioning balls need to be placed on the measured object, which is obviously not applicable for full inspection or on-line measurement. The most commonly-used optimal algorithm is the ICP algorithm, and many scholars have studied it. The ICP method was presented by Besl and McKay [34]. The goal of this method is to obtain an accurate solution by minimizing the distance between point-correspondences, known as the closest point. According to the strategy of determining the corresponding point pair, a variety of optimization algorithms has evolved [35,36,37,38], and the main bottlenecks of these algorithms are the search and optimization calculation of the corresponding point pair and being more sensitive to the initialization location of the point cloud and CAD model; especially for objects that do not have obvious geometric features, the convergence effect is not ideal. In order to achieve higher registration accuracy, the iteration times are more, which is more time consuming.

In this paper, the line structure optical scanning method is adopted to obtain the rail surface point cloud, which is of high precision, fast and convenient. On the basis of the conventional ICP algorithm, the Kalman filter model [39,40,41] is used to predict the transformation of the current profile through the recursion of the adjacent continuous profiles, which greatly reduces the iteration times. At last, the optimal registration parameters are selected by synthetically evaluating segmented ICP and overall ICP. 

## 3. Design of the 3D-LPS

### 3.1. System Principle

**Figure 1:**
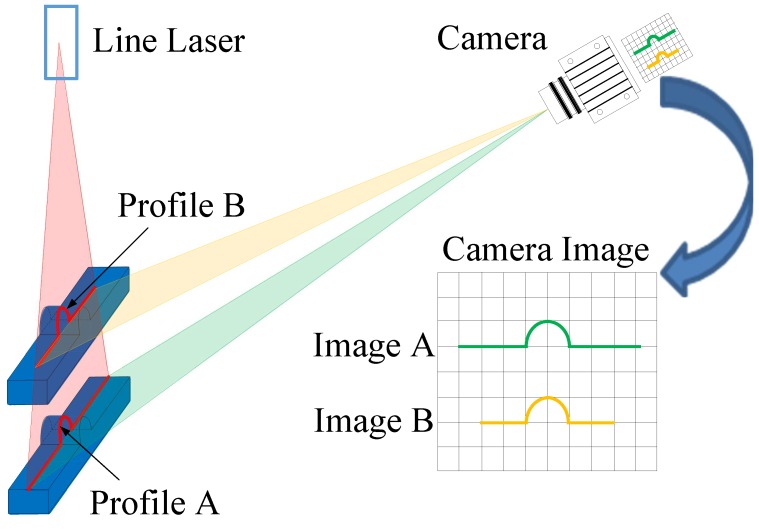
System principle.

The key technologies of 3D surface defect detection include the 3D data acquisition and 3D point cloud processing aspects. Research works in 3D surface defect detection are mainly concentrated on the 3D visual sensors and data registration between the 3D point cloud and CAD model. In the rail surface defect detection, the first step is to obtain surface point cloud data. As the line structure optical scanner has been applied to many aspects of surface defect detection, this system adopts the Keyence LJ-V7000 type profilometer for rail surface profile acquisition. The measurement principle is shown in Figure 1.

In Figure 1, the line laser irradiates vertically and forms a light band on the surface of the object to be measured. The camera is tilted at an angle with respect to the line laser and takes a photo of the object surface. Then, the light band on the surface will reflect the object surface profile in the laser projection surface. In the situation that the relative position relationship between the camera and the line laser is determined and unchanged, for the same object profile, it can be seen that the image’s position of the light band on the camera (Image A to Image B) will change with the object’s depth (Profile A to Profile B) outside. Therefore, by detecting the position and shape of the light band’s image on the camera, the physical coordinates of the light band are calculated, that is to say the physical coordinates of the surface profile are calculated. With the object not moving, the carrier platform moves along and divides the measured object surface into many profiles, so as to achieve the goal of measuring the physical coordinates of all of the surface points.

### 3.2. System Architecture

**Figure 2:**
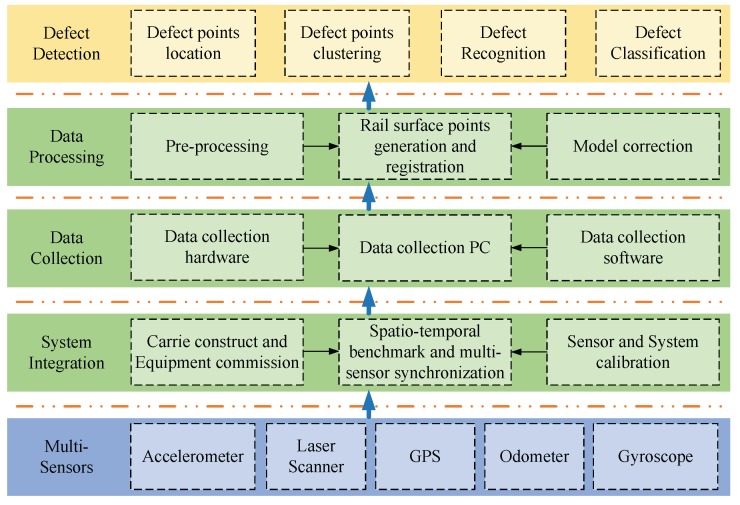
System architecture.

According to the discussion above, once the rail surface profile is obtained, the deviation between the defective surface and the standard model can be calculated. To achieve the goal of detecting the whole surface defects, continuous measurement should be taken. Thus, several different kinds of sensors are integrated into one whole system, e.g., laser scanner, odometer, IMU, GPS, etc. The architecture of 3D-LPS is shown in Figure 2. It can be seen that the system is highlighted at five levels. The multi-sensors level lists all of the sensors involved in the 3D-LPS. The system-integration level is responsible for the synchronization of the whole system under a unified spatio-temporal benchmark. On this level, each sensor is fixed on the carrier platform, and calibration of each sensor should be done. The data-collection level connects the hardware and the software to capture various data in a computer. Once the data have been collected, the data processing level fuses the raw data to extract the 3D point cloud of a rail surface. Through data registration with the standard model, the defect-detection level analyses the deviation between the defective rail surface and the standard rail model and hence locates and recognizes the defects.

**Figure 3:**
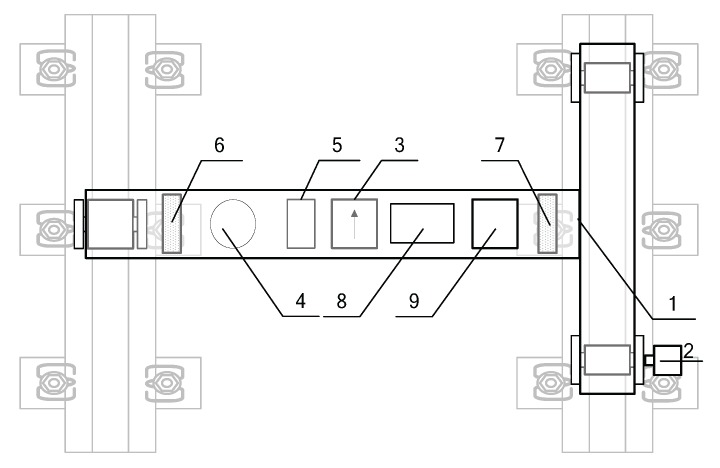
System components’ arrangement.

In 3D-LPS, the arrangement of each part is shown in Figure 3: the laser scanners (6,7) are installed on the side of the platform (1) to obtain the rail surface profile; the odometer (2) is equipped on the wheel and collects the distance and speed data of the platform; the GPS (4) is fixed above the platform and records the platform’s location data; the IMU (3) is mounted on the middle of the platform gaining the platform’s attitude data; the synchronization control unit (5) provides unified spatio-temporal benchmark; all of the sensor data and synchronous data are transferred to the computer (8) for fusion processing; the power-supply module (9) is placed on the platform to supply power for all equipment and sensors.

### 3.3. Pre-Processing

The rail surface profile data collected by the laser scanners cannot be directly used for the extraction of rail surface defect and needs to be pre-processed. Firstly, a motion correction model is designed to correct the error caused by slight rotation of the platform. Then, each sensor’s coordinate should be unified to the same coordinate system, where the IMU coordinate system is taken as the reference coordinate system.

**Figure 4:**
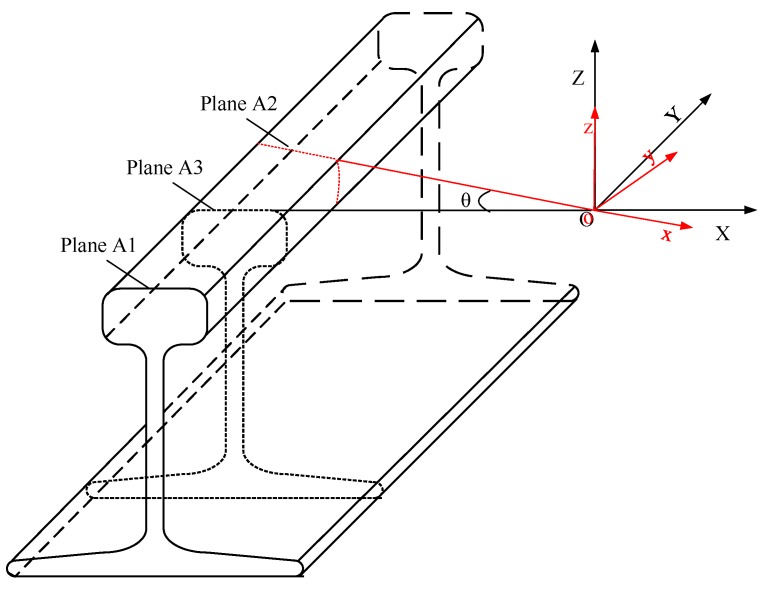
Motion correction model.

Motion correction model: As shown in Figure 4, the platform body is supported by three wheels on the rail surface. During the movement of the platform, the three support points would not leave the rail top; therefore, it is reasonable to assume that only horizontal rotation of the IMU exists between adjacent profiles. AT this point, if the platform has slight rotation, it may cause the profile captured by the laser scanner not to be perpendicular to the rail, which cannot match the standard rail model.

Suppose the rail surface profile gained at time $t_{1}$ is $A_{1}$ as shown in Figure 4; at time $t_{2}$, the platform moves forward and obtains profile $A_{2}$; the rotation angle between the adjacent profile $A_{1}$ and $A_{2}$ is θ; if the correct profile at time $t_{2}$ is supposed to be $A_{3}$, then $A_{3}$ will be parallel to $A_{1}$, so $A_{3}$ is the projection of $A_{2}$ on $A_{1}$. Thus, suppose the original point coordinate of the left and right rail surface profile is $P_{L0}$, $P_{R0}$; the corrected point coordinate of left and right rail surface profile will be Equations (1) and (2).

(1) $$P_{L}=P_{L0}\left[\begin{array}{ccc} \cos\theta & -\sin\theta & 0\\ \sin\theta & \cos\theta & 0\\ 0 & 0 & 1 \end{array}\right]$$

(2) $$P_{R}=P_{R0}\left[\begin{array}{ccc} \cos\theta & -\sin\theta & 0\\ \sin\theta & \cos\theta & 0\\ 0 & 0 & 1 \end{array}\right]$$

Unified coordinate transformation: Choose the IMU coordinate system as the reference coordinate system; suppose the coordinate of the left laser scanner coordinate center in the referenced coordinate system is $\left(R_{L}^{I},T_{L}^{I}\right)$; the same, the right laser scanner coordinate center is $\left(R_{R}^{I},T_{R}^{I}\right)$; then, the coordinate of the left and right rail surface profile in the referenced coordinate system can be expressed as Equations (3) and (4).

(3) $$P_{L}^{I}=R_{L}^{I}P_{L}+T_{L}^{I}$$

(4) $$P_{R}^{I}=R_{R}^{I}P_{R}+T_{R}^{I}$$

In practical work, as the IMU moves along the rail direction, the trajectory of the IMU coordinate origin can be calculated by the GPS’s location, the IMU’s attitude and the odometer’s mileage. Therefore, the pre-processed point cloud reflects the relative position of the actual rail surface points.

### 3.4. Calibration of 3D-LPS

**Figure 5:**
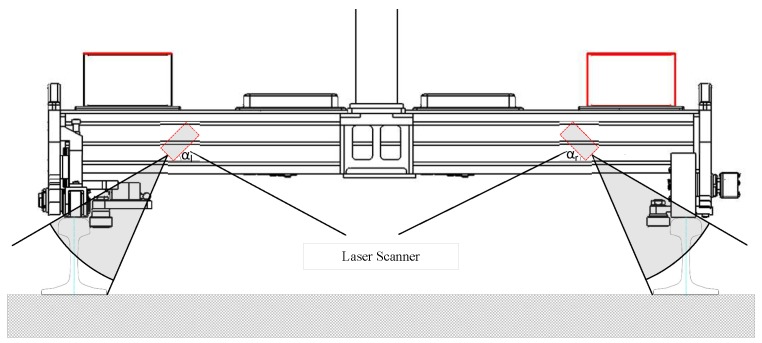
System calibration.

The 3D-LPS needs to be calibrated before actual measurement. The main work of calibration is to find out the transformation parameters of the left and right laser scanner coordinate center in the referenced coordinate system, $\left(R_{L}^{I},T_{L}^{I}\right)$ and $\left(R_{R}^{I},T_{R}^{I}\right)$. As shown in Figure 5, the left laser scanner has a right deflection angle of $\alpha_{l}$, scanning the inner side of the left rail. The right laser scanner has a left deflection angle of $\alpha_{r}$, scanning the inner side of the right rail.

The specific calibration steps are:

(i) Choose the center of the left rail top as the coordinate origin; use the gauging rule measuring the gauge *G* between the left and right rail; measure the distance from the center of the right rail top to the center of the left rail top; record as *D*;
(ii) According to *D* and the standard rail model, let the center of the left standard rail model top be the coordinate origin, then the center of the right standard rail model top will be located on (*D*, 0);
(iii) Let the structured light vertically project onto the rail top and capture the rail surface profile;
(iv) Adjust the left rail surface profile manually until it completely matches with the left standard rail model; calculate the transformation parameters as $\left(R_{L}^{I},T_{L}^{I}\right)$; the same for $\left(R_{R}^{I},T_{R}^{I}\right)$.

## 4. Rail Surface Defect Detection Method

After pre-processing, the rail surface point cloud is unified into the same coordinate system, and it should be matched with the standard rail model to calculate the rail defects. This section introduces the ICP algorithm and points out the problem of ICP, then proposes an AICP algorithm based on the Kalman filter model, which improves the registration accuracy to the sub-millimeter level, so as to locate the defect area accurately. On this basis, the k-means algorithm [42,43,44] is used to cluster the defects and extract the characteristics of each defect area; at last, the decision tree classifier is taken to identify the type of rail surface defect.

### 4.1. Rail Profile Registration

ICP: For the 3D point set registration problem, the most widely-used and the most influential one is the ICP algorithm proposed by Besl and Mckay in 1992. The ICP algorithm is a 3D object alignment algorithm based on a pure geometric model; its essence is the optimal matching algorithm based on the least squares method. It repeats the process of first determining the set of corresponding relationships and then calculating the optimal rigid transformation until a convergence criterion of correct matching is met, so that the optimal match of the two matching data satisfies the certain degree of metric, so as to find the the translation transformation *T* and rotation transformation *R* between the target point set *P* and the reference point set *Q*. Suppose the target point set is $\left\{P_{i},i=1,2\ldots \right\}$, and the reference point set is $\left\{Q_{i},i=1,2\ldots \right\}$; in the *k*-th iteration, find the point set $\left\{Q_{i}^{k},i=1,2\ldots \right\}$ from *Q* that corresponds with target point set *P*, then calculate the transformation matrix between *P* and $\left\{Q_{i}^{k},i=1,2\ldots \right\}$, and update the original point set until the average distance between the dataset is less than the given τ; that is to say, satisfy the following formula $f\left(R,T\right)=\sum_{i=1}^{n}{∥Q_{i}-\left(RP_{i}+T\right)∥}^{2}=min$. The specific steps are:

(i) Take point set $P_{i}^{k}$ from target point set *P*;
(ii) Calculate the point set $Q_{i}^{k}$ from reference point set *Q* as the corresponding point set of $P_{i}^{k}$, and make $∥Q_{i}^{k}-P_{i}^{k}∥=min$;
(iii) Calculate the transformation from $P_{i}^{k}$ to $Q_{i}^{k}$, and make $∥Q_{i}^{k}-P_{i}^{k}∥=min$, then record the rotation vector as $R^{k}$ and translation vector as $T^{k}$;
(iv) Update the point set, and compute $P_{i}^{k+1}=R^{k}P_{i}^{k}+T^{k}$;
(v) Calculate the average distance between $P_{i}^{k}$ and $Q_{i}^{k}$, and record as $d^{k+1}=\frac{1}{n}\sum_{i=1}^{n}{∥P_{i}^{k+1}-Q_{i}^{k}∥}^{2}$;
(vi) If $d^{k+1}\geq \tau$, then return to Step 2 until $d^{k+1}<\tau$ or the iteration times are greater than the preset maximum iteration times.

The ICP algorithm has a good effect on point set registration; however, in this application, there is sometimes a big difference between the defective rail surface point cloud and the standard rail model. For example, if the abrasion wear is serious, or the scratches are deep, then the defective rail surface point cloud would be very different from the standard rail model, resulting in poor registration and even matching errors. That is why it is necessary to improve the ICP algorithm to match the rail surface point cloud registration.

AICP: Generally, the rail top and rail side are easily abraded and scratched, which usually causes the defective rail surface, different from the standard model. The bottom of the rail only has corrosion because of no contact with the wheel, but the corrosion will not make the rail bottom’s shape very different from the standard model, so this is an important reference of the data registration afterwards. At the same time, when the system is moving along the rail, it obtains the rail surface profile every 1 mm. In such a small interval, there is not enough time for the system’s attitude to change suddenly when obtaining adjacent profiles. Thus, the transformation between the current rail surface profile and the standard model is closely related to the transformation between the adjacent profile and the standard model. On this basis, this paper proposes an AICP algorithm, using the Kalman filter model to predict the transformation of the current profile through the recursion of the adjacent continuous profiles and then calculating the average distance between each part of point cloud and the standard point cloud under this transformation parameter and, at last, choosing the corresponding optimal transformation parameter by evaluating these average distances. The specific steps are:

(i) Suppose the *N*-th profile point set is $P^{N}$, and take the transformation (translation $T^{N-1}$ and rotation $R^{N-1}$) between the (N−1)-th profile point set $P^{N-1}$ and the standard model $Q_{M}$ as the predicted transformation from $P^{N}$ to $Q_{M}$;
(ii) Divide the rail surface profile into two parts: the rail head part and rail bottom part. Pay attention that the rail head is different from the rail head clarified by Lin; the rail head here is just part of the whole rail head and is the upper part of the profile points, which is used to distinguish from the bottom part of the profile points, the rail head point set $P_{H}^{N}$ and the rail bottom point set $P_{B}^{N}$, with the $\left(R^{N-1},T^{N-1}\right)$ as the initial value. Calculate the transformation from $P_{H}^{N}$, $P_{B}^{N}$ to $Q_{M}$ separately, and record as $\left(R_{H}^{N},T_{H}^{N}\right)$, $\left(R_{B}^{N},T_{B}^{N}\right)$, then output each part’s average distance $d_{H}^{N}$, $d_{B}^{N}$;
(iii) Calculate the minimum value of $d_{H}^{N},d_{B}^{N}$, and take the transformation corresponding with the minimum value as the calculated transformation from $P^{N}$ to $Q_{M}$;
(iv) Calculate the average distance $d^{N}$ from $P^{N}$ to $Q_{M}$ under the transformation parameter $\left(R^{N-1},T^{N-1}\right)$;
(v) Evaluate the $min(d_{H}^{N},d_{B}^{N})$ and $d^{N}$; the greater the distance, the smaller the weight and the smaller the probability. Update the probability $p^{N-1}=\frac{d^{N}}{\sqrt{{\left(min\left(d_{H}^{N},d_{B}^{N}\right)\right)}^{2}+{\left(d^{N}\right)}^{2}}}$ and $p^{{}^{\prime }N}=\frac{min(d_{H}^{N},d_{B}^{N})}{\sqrt{{\left(min\left(d_{H}^{N},d_{B}^{N}\right)\right)}^{2}+{\left(d^{N}\right)}^{2}}}$;
(vi) According to the Kalman filter model, update the transformation between $P^{N}$ and $Q_{M}$ as $\left(R^{N},T^{N}\right)=p^{N-1}\left(R^{N-1},T^{N-1}\right)+p^{{}^{\prime }N}\left(R^{{}^{\prime }N},T^{{}^{\prime }N}\right)$.

### 4.2. Rail Defect Detection

**Figure 6:**
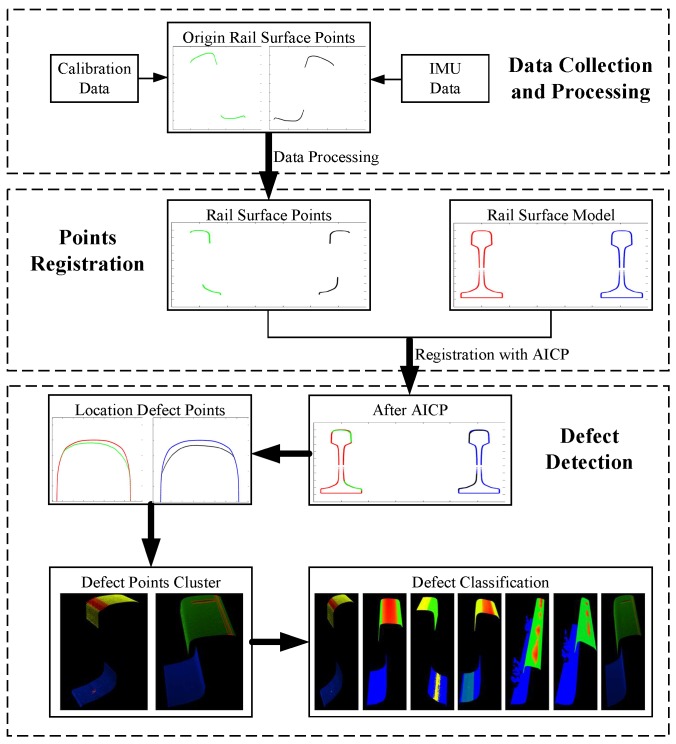
Defect detection and classification procedure.

As shown in Figure 6, after being processed by AICP, the point set registration accuracy is down to the sub-millimeter level. It is easy to locate the candidate defective area accurately by comparing the deviation of registered rail surface profile and the standard model with the given threshold. By combining the continuous defective profiles together, the candidate defect points are merged into candidate defect regions. The minimum circumscribed rectangle and center of each defect region are calculated for the using the k-means clustering, which connects the small defect region with large defect area and clusters the defect regions with similar characteristic together. At last, the location, shape, depth, length, width, gradient, minimum circumscribed rectangle and other geography characteristics can be calculated. The specific steps are:

(i) Suppose the current profile is $P=\{P_{i}|i=1,2\ldots n\}$ after registration. Let *n* stand for the point number in current profile, and let the standard model be $Q_{M}=\left\{Q_{j}|i=1,2\ldots m\right\}$. For every point $P_{i}$, take its x coordinate as the reference, and choose the point in $Q_{M}$ that has the closest x coordinate value with $P_{i}$ as $P_{i}$’s corresponding point, then pick out *P*’s corresponding point set $Q=\{Q_{i}|i=1,2\ldots n\}$ in $Q_{M}$;
(ii) Make the deviation of *Q* and *P*, and calculate the deviation set $D=\{D_{i}=Q_{i}-P_{i}|i=1,2\ldots n\}$;
(iii) Let $\tau_{D}$ be the depth threshold of the rail surface defect, and pick out the point in *P* that has a deviation value in *D* greater than $\tau_{D}$ as the defect point, then gather these defect points as the current profile’s defect point set $C=\{D_{k}|D_{k}>\tau_{D},1<k<n\}$; *k* means the defect point number in the current profile;
(iv) Take continuous defective profiles as a defect point set $PD=PD_{st}|s=1,2\ldots N;t=1,2\ldots s_{k}$, and let $s_{k}$ be the defect point number of the *s*-th profile
(v) Use k-means method to cluster all of the defect point sets, and divide into *M* bounding boxes $DB=DB_{m}|m=1,2\ldots M$; each box would contain several defect point set. Record its number $N_{m}$, and calculate its center $C_{m}$, radius $R_{m}$ and distance between different defect point sets $D_{m}$;

According to the process above, Step 3 defines a depth threshold $\tau_{D}$, which is an empirical value. For rails in service, their head parts have more or less abrasion, so $\tau_{D}$ is necessary to highlight serious defects and is mainly determined by rail service time. In this step, $\tau_{D}$ takes a value of 1 mm on the rail head and 0.5 mm on the rail bottom. Step 4 obtains many small defects. In this step, the location, depth, length, width, gradient, minimum circumscribed rectangle can be figured out. Step 5 connects some defects together to provide regional characteristics, for example the center, radius, maximum class separation distance, minimum class separation distance, and so on. All of these characteristics help to achieve defect detection and provide effective information for defect classification.

### 4.3. Rail Defect Classification

**Figure 7:**
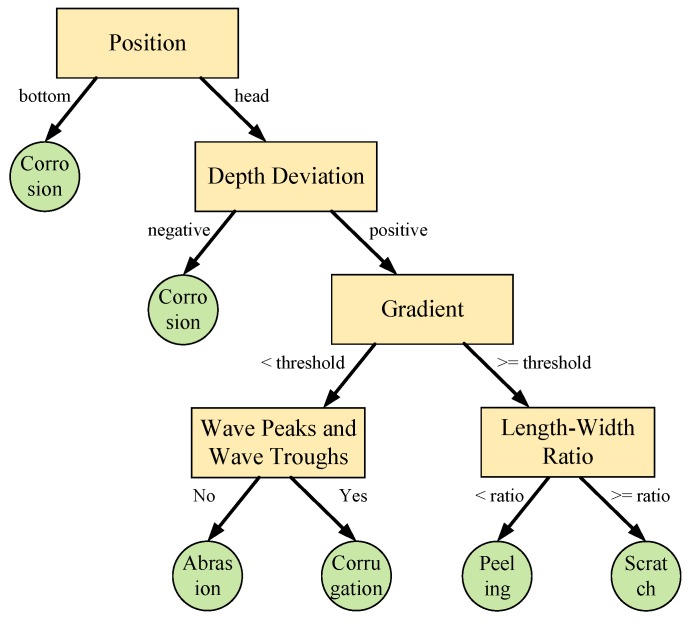
Decision tree construction.

According to the discussion above, different defects have different characteristics. In general, the train wheel contacts and rubs against the rail head, which causes abrasion, corrugation, scratch and peeling. Therefore, the corrosion on the rail bottom could be distinguished by position, but corrosion on the rail head is still mixed with other defects. As we know, the friction will cause the rail head (the part contacting the train wheel) to be lower than the standard rail model, then the depth deviation for the rail model is positive. The other way around, the corrosion on the rail head will return to a negative depth deviation, which differentiates the rail head corrosion from the other four defects. Among the four classes of defect, abrasion and corrugation have a smooth profile, but scratching and peeling have sharp edges, so the difference is reflected in the gradient. Abrasion and corrugation can be distinguished by wave peaks and wave troughs. Scratching is always long and narrow, that is to say the length-width ratio is bigger than peeling. Based on these judgments, the decision tree (DT) [45,46,47] is adopted to classify the defect and constructed as follows in Figure 7.

## 5. Experiments and Results

**Figure 8:**
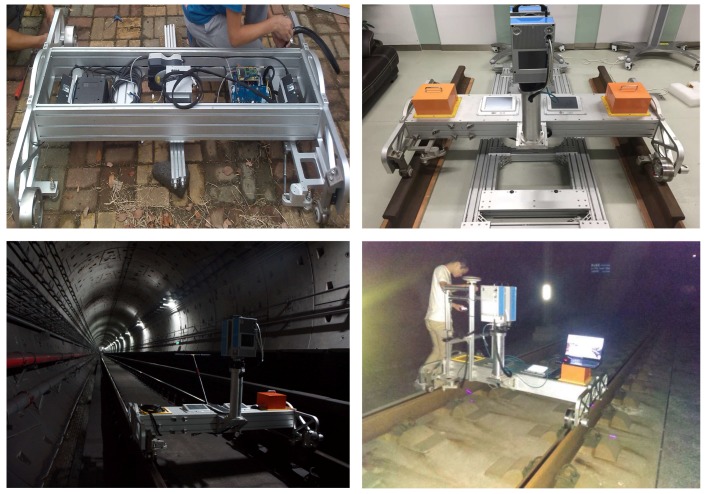
Experiment environment.

The 3D-LPS is jointly developed by Wuhan University and Shenzhen University. In order to verify its performance, a series of experiments indoors and outdoors is performed. Some of the experimental environment is shown in Figure 8. The parameters of each sensor are selected as: the odometer installation wheel circumference is 500 mm, and the odometer’s frequency is 500 Hz, which means the odometer will output a pulse every 1 mm. The pulse triggers the laser scanner to capture a profile. Eight hundred points are sampled on one profile. The accuracy in the width direction is 0.3 mm and in the height direction is 0.01 mm. The IMU has an attitude accuracy of $0.02^{\circ }$ and an azimuth accuracy of $0.05^{\circ }$. The platform moves at a speed of about 1.5 m/s.

In order to evaluate the accuracy of the calibration, The static collected dataset Data 1 is constructed to verify the unilateral and bilateral precision, respectively. For the purpose of verifying the matching effect of the proposed AICP algorithm, the dynamic collected dataset Data 2 is constructed to compare with ICP in terms of iteration times, calculation time, bias after registration and the matching accuracy. In order to verify the effectiveness of the defect detection algorithm, an indoor short-range defective marker dataset Data 3 is constructed to compare the recognition results with the ground truth results. An outdoor long-range rail defect dataset Data 4 is collected to compare the classification results with the results obtained by human vision.

### 5.1. System Calibration Accuracy Verification

Data 1 is used to verify the accuracy of system calibration. The calibration is a critical component of a measurement system [48]. For the Wuhan high-speed rail, ten groups of rail surface profiles are obtained in different sleepers, and the gauge of the sleeper is measured by a gauging rule. The rail with a specification of 60 kg/m is used, and it has a standard gauge of 1435 mm. Under the calibration parameters, we calculate the average distance $D_{HM}$ between the rail head surface profile and the standard rail model, the average distance $D_{BM}$ between the rail bottom surface profile and the standard rail model to evaluate the calibration accuracy of unilateral laser sensor and the IMU center. We calculate the gauge of the left rail profile and the right rail profile $G_{P}$ and compare it with the gauge measured by the gauging rule to evaluate the calibration accuracy of the left laser sensor and the right laser sensor. The results are shown in Table 1.

It can be seen from the results that, under the calibration parameters, the rail profiles measured in different places on the rail are well coincident with the standard rail model. From Table 1, the deviation between the gauge calculated under the calibration parameter and the gauge measured by the gauging rule proves the accuracy of the system calibration. The average distance between the rail head surface profile and the standard rail model is about 1–2 mm, which is larger than the average distance between the rail bottom surface profile and the standard rail model. This is reasonable because the rail used in the experiment has only been in service for a short time, and the rail head has a slightly abrasion, while the right side rail head has a deeper abrasion than the left side. Meanwhile, the inner side of the rail was abraded more seriously. Even with the above complications, the system calibration still achieves an accuracy of sub-millimeters.

### 5.2. Comparison of AICP with ICP

Data 2 is used to verify the efficiency and effectiveness of the AICP algorithm. For Shenzhen Metro Line 1, a 400 m-long rail is profiled dynamically, and four typical rail sections are selected. The first section has very few defects. The second section has some defects on the rail head. The third section has some defects on the rail bottom. The forth section has some defects on both the rail head and the rail bottom. We choose 1000 profiles for each section manually and compute the statistics value to study the difference. The result is listed in Table 2. The iteration times IT, the calculation time CT, the total calculation time TT, the accuracy rate of registration AR and the average distance *D* of the ICP and AICP are calculated, which are listed in Table 2. In Table 2, the unit for CT and TT is seconds, and the unit for *D* is millimeters; the subscript F denotes the full rail profile; H denotes the head part; and B denotes the bottom part.

**Figure 9:**
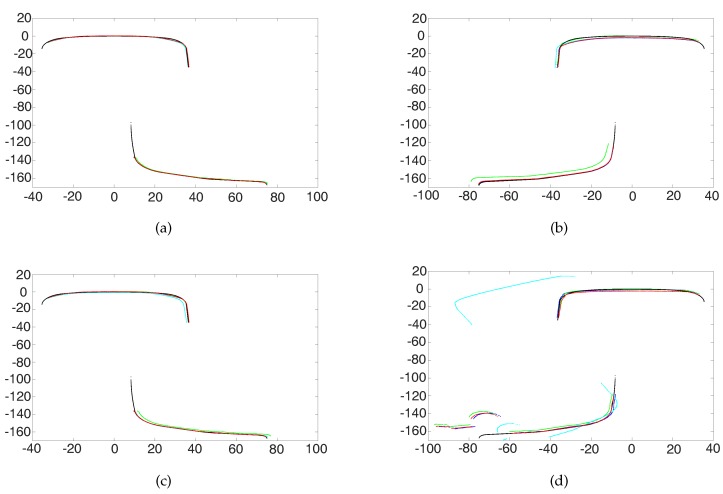
Result of ICP and AICP: (**a**) With less defects; (**b**) With defects on the rail head; (**c**) With defects on the rail bottom; (**d**) With defects on both the head and bottom.

Some examples are shown in Figure 9, where the black point set denotes the standard rail model, the blue point set denotes the full rail profile ICP matching result, the green point set denotes the results of rail head part in ICP matching, the cyan point set denotes the results of the rail bottom part in ICP matching and the red point set denotes the AICP algorithm matching results. As shown in the "*TT*" column of Table 2, the AICP algorithm is faster than the ICP algorithm. In Group 1, the measured profiles have a few defects as shown in Figure 9a, and the results of ICP are similar to AICP on both the rail head and the rail bottom, shown in the *D* columns. In Group 2, comparing the $D_{H}$ column and the $D_{B}$ column, we can see that if taking the rail head part as the reference, $D_{H}$ is much bigger, which indicates that there are defects on the rail head part, as shown in Figure 9b. From the blue parts, we can see that the rail head has an abrasion of about 1 mm. When taking the head part as the reference (the green part), the bottom part is obviously not correctly matched to the model bottom. In Group 3, comparing the $D_{H}$ column and the $D_{B}$ column, we can see that if taking the rail bottom part as the reference, $D_{B}$ is much bigger, which indicates that there are defects on the rail bottom part, as shown in Figure 9c. From the blue parts, we can see that there is corrosion on the rail bottom. When taking the bottom part as the reference (the cyan), the head part is obviously not correctly matched to the model head. In Group 4, evaluating the $D_{H}$ column and the $D_{B}$ column, we can see that if taking the rail head part as the reference, $D_{H}$ and $D_{B}$ are both much bigger, which indicates that there are defects on the both rail head part and the rail bottom part, as shown in Figure 9d, where the blue parts themselves do not match the model. Let us take the red part as the best matched transform; we can see that the rail head has abrasion on the top and the side, and the rail bottom has obvious changes from the rail model. In this case, the matching results of ICP with the rail head as the reference are bad, and the matching results with the rail bottom as the reference are totally wrong. From the comparison results, we can conclude that the proposed AICP is much faster and more effective than the ICP, and it brings good matching results in each case.

### 5.3. Rail Surface Defect Extraction

Data 3 is used to evaluate the defect-extraction performance of the proposed algorithm. A 1.5-m rail is placed indoors, and 10 artificial defects are made. We measure and record the length and maximum depth of each defect with a micrometer. We scan all of the defects and repeat 10 times. We compare the defect length (that is, along the direction of rail extension) Len, the defect width (that is, perpendicular to the direction of rail extension) Wid and the maximum depth $D_{max}$ extracted by the 3D-LPS with the actual length Length, actual width Width and the actual maximum depth Depth measured by human workers. In this procedure, the k-means clustering algorithm takes k = 5 to divide the defective region every 100 profiles (100 mm long). The center of the longest continuous defective region is chosen to be the original cluster center; thus, two cluster centers (one on the rail top and the other on the rail bottom) can be quickly computed. The other three centers are randomly chosen outside of these two continuous regions. In this way, the computation time is proven to be greatly shortened. The results are shown in Table 3.

**Figure 10:**
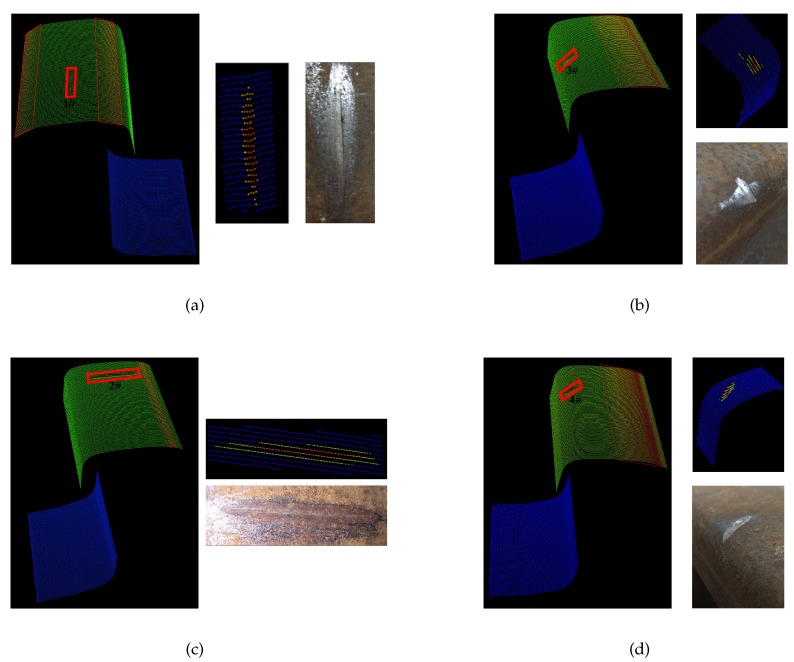
Examples of typical artificial defect extraction: (**a**) Vertical defects on the top; (**b**) Horizontal defects on the edge; (**c**) Horizontal defects on the top; (**d**) Sloping defects on the edge.

As shown in Table 3, 10 typical artificial defect areas are made and measured. Groups 1–3 are the result of defect extraction of vertical defects on the rail top, as shown in Figure 10a. Groups 4–6 are the result of defect extraction of horizontal defects on the rail top, as shown Figure 10c. Groups 7 and 8 are the result of defect extraction of horizontal defects on the rail edge, as shown in Figure 10b. Groups 9 and 10 are the result of defect extraction of sloping defects on the rail edge, as shown in Figure 10d. Experimental results show that the proposed algorithm is very sensitive to defects with an obvious change in depth and can locate the defects accurately with a millimeter length accuracy and a sub-millimeter depth accuracy. To be more exact, the system can recognize defects with length (along the direction of the rail extension) larger than 2 mm, width (perpendicular to the direction of the rail extension) larger than 0.6 mm and depth larger than 0.5 mm. The algorithm is less sensitive to changes in length, and the defect area detected is larger than the ground truth. This is mainly because the defect depth of the edge area is too small (sub-millimeter level) to be observed by human vision. In summary, the proposed algorithm is effective at detecting defects with an obvious change in depth.

### 5.4. Result of Defect Classification

Data 4 is used to validate the defect classification accuracy of the 3D-LPS. For the Wuhan high-speed rail, Shenzhen Metro Line 1 and indoor rail, 450 defects (150 abrasion, 80 corrugation, 50 scratch, 120 corrosion, 50 peeling) are collected as a dataset to validate the accuracy of the classification module. The rail defects are extracted from the rail surface point cloud and classified into five categories: abrasion, corrugation, scratch, corrosion and peeling. The ground-truth types of the defective area are identified by human eyes in the point cloud using the visualization software CloudCompare. The results are shown in Table 4.

Table 4 shows the confusion matrix of the classification results obtained by our decision tree classifier (DT). The confusion matrix is used to evaluate the classification accuracy and reliability. From Table 4, we can see that 422 (141 + 74 + 43 + 120 + 44) defects are correctly classified, which denotes an overall accuracy of 93.78%. The corrosion classification accuracy is up to 100%, which is because the position (head or bottom) and depth deviations (positive or negative) are accurate and unique. The abrasion and corrugation are less correctly classified. A possible reason is that the choice of the depth deviation threshold is not robust enough. The scratching and peeling are two types that are easily confused (two scratches incorrectly classified as peeling and three peelings as scratches) due to the fact that the gradient threshold is hard to set. Another important reason for incorrect classification is that some defects are the mixture of multiple types of defects. For example, scratching or peeling can exist in abrasion or corrugation (in this test, 11 scratches and nine peelings coexist with abrasion and corrugation, but 5 scratches, 3 peelings and 3 abrasions are classified incorrectly, the miss-classification ratio coming from coexisting defects is up to 55%), and we will try to solve this problem in our future work. Overall, the experimental results show that the proposed classification algorithm is effective and reliable. 

## 6. Conclusions

This paper introduced a 3D laser profiling system for rail surface defect detection to acquire rail surface’s depth data accurately and efficiently and proposed an AICP algorithm based on the Kalman filter model. The improved ICP algorithm takes into account the characteristics that the attitude of adjacent profiles changes smoothly and slowly, and the initial value of ICP for the current profile is predicted by the adjacent continuous profiles, which greatly improves the convergence speed. On the other hand, partly registration was calculated to select the optimal registration parameters, and the Kalman filter model was adopted to update the final optimal registration parameters, which solves the problem of the wrong match in the ICP algorithm. Using the proposed AICP algorithm, the registration achieved an accuracy of sub-millimeters and made possible the accurate location of defects. In addition, the k-means algorithm was employed to cluster the defects and extract the characteristics of each defect area, based on which a decision tree classifier was applied to identify the type of rail surface defect. The experimental results showed that the proposed algorithm can register the rail surface profile with the standard rail model quickly and accurately and locate the defect area correctly, as well as achieving a good performance on defect classification. In our future work, we will try other learning algorithm, such as SVM [49,50], to train the classifier to further improve the classification accuracy.

## Figures and Tables

**Table 1:** System calibration accuracy.

ID	$D_{\mathrm{HM}}^{L}$ (mm)	$D_{\mathrm{HM}}^{R}$ (mm)	$D_{\mathrm{BM}}^{L}$ (mm)	$D_{\mathrm{BM}}^{R}$ (mm)	$G_{P}$ (mm)	$G_{M}$ (mm)
1	0.95	0.53	1.95	1.41	1435.34	1435.42
2	0.86	0.51	1.89	1.53	1435.46	1435.57
3	0.63	0.54	1.81	1.24	1435.81	1436.02
4	0.72	0.41	1.74	1.28	1436.35	1436.29
5	0.78	0.38	1.75	1.32	1436.19	1436.24
6	0.91	0.61	1.79	1.43	1436.25	1436.37
7	0.87	0.42	1.71	1.35	1435.78	1436.12
8	0.76	0.58	1.85	1.38	1435.49	1435.42
9	0.92	0.49	1.91	1.41	1435.81	1435.64
10	1.12	0.64	1.95	1.49	1436.06	1436.31

**Table 2:** Effect of AICP and ICP.

ID	Type	Statistical value	${\mathrm{IT}}_{F}$	${\mathrm{CT}}_{F}$	$D_{F}$	${\mathrm{IT}}_{H}$	${\mathrm{CT}}_{H}$	$D_{H}$	${\mathrm{IT}}_{B}$	${\mathrm{CT}}_{B}$	$D_{B}$	TT	*AR*
1	ICP	Average value	26.8	0.267	0.273	55.6	0.173	0.196	48.3	0.163	0.067	0.603	99.9%
		Standard deviation	2.21	0.048	0.011	3.22	0.018	0.012	3.64	0.024	0.006	-	-
		Maximum value	30	0.346	0.291	60	0.198	0.213	54	0.203	0.075	-	-
	AICP	Average value	22.4	0.238	0.273	35.1	0.117	0.196	48.2	0.161	0.067	0.516	99.9%
		Standard deviation	1.02	0.006	0.012	1.45	0. 003	0.014	2.74	0.022	0.006	-	-
		Maximum value	24	0.246	0.293	37	0.121	0.217	52	0.198	0.075	-	-
2	ICP	Average value	25.8	0.264	0.423	51.3	0.148	0.361	46.2	0.162	0.069	0.574	33.3%
		Standard deviation	2.24	0.045	0.012	5.62	0.024	0.025	3.12	0.022	0.007	-	-
		Maximum value	29	0.338	0.441	61	0.186	0.395	50	0.192	0.081	-	-
	AICP	Average value	24.6	0.241	0.424	33.5	0.119	0.364	49.6	0.164	0.068	0.525	99.9%
		Standard deviation	1.84	0.018	0.012	3.23	0.005	0. 026	2.81	0.018	0.006	-	-
		Maximum value	27	0.268	0.443	38	0.127	0.396	53	0.191	0.077	-	-
3	ICP	Average value	27.8	0.322	0.418	30.6	0.109	0.201	74.8	0.297	0.157	0.728	33.4%
		Standard deviation	2.31	0.052	0.013	3.21	0.008	0.015	5.47	0.017	0.011	-	-
		Maximum value	31	0.407	0.442	35	0.121	0.223	82	0.323	0.173	-	-
	AICP	Average value	14.5	0.188	0.418	45.2	0.139	0.201	24.3	0.109	0.155	0.436	99.9%
		Standard deviation	4.67	0.038	0.014	3.18	0.016	0.013	6.74	0.011	0.011	-	-
		Maximum value	22	0.237	0.445	50	0.168	0.219	35	0.127	0.175	-	-
4	ICP	Average value	21.6	0.237	0.566	53.4	0.167	0.471	53.6	0.306	0.408	0.721	0.01%
		Standard deviation	6.43	0.074	0.035	4.36	0.025	0.023	7.01	0.049	0.015	-	-
		Maximum value	30	0.357	0.621	60	0.205	0.503	63	0.382	0.445	-	-
	AICP	Average value	26.7	0.257	0.568	30.2	0.109	0.472	16.8	0.129	0.098	0.495	99.9%
		Standard deviation	3.21	0.021	0.029	3.43	0.008	0.016	4.92	0.018	0.013	-	-
		Maximum value	32.	0.288	0.618	35	0.122	0.496	24	0.158	0.117	-	-

**Table 3:** Result of rail surface defect extraction.

ID	*Len* (mm)	*Length* (mm)	*Wid* (mm)	*Width* (mm)	$D_{\max}$ (mm)	*Depth* (mm)
1	22	22.76	1.5	1.56	1.7745	1.80
2	15	15.54	1.2	1.24	0.8174	0.84
3	18	18.18	1.8	1.78	1.3548	1.36
4	4	4.08	32.7	32.64	0.6854	0.66
5	4	4.12	26.4	26.22	0.8123	0.80
6	5	4.96	15.3	15.12	1.0254	1.08
7	5	4.92	7.8	7.76	1.1214	1.12
8	5	4.94	10.2	10.24	1.0146	0.98
9	6	6.08	9.3	9.38	0.8564	0.86
10	6	6.12	10.5	10.68	0.9631	0.96

**Table 4:** Classification result by DT.

Defect Type		Predicted					
		Abrasion	Corrugation	Scratch	Corrosion	Peeling	Total
**Actual**	**Abrasion**	141	6	2	0	1	150
	**Corrugation**	6	74	0	0	0	80
	**Scratch**	3	2	43	0	2	50
	**Corrosion**	0	0	0	120	0	120
	**Peeling**	2	1	3	0	44	50

## References

[1] Kumar A., Pang G.K. (2002). Defect detection in textured materials using Gabor filters. IEEE Trans. Ind. Appl..

[2] Toliyat H.A., Abbaszadeh K., Rahimian M.M., Olson L.E. (2003). Rail defect diagnosis using wavelet packet decomposition. IEEE Trans. Ind. Appl..

[3] Mandriota C., Nitti M., Ancona N., Stella E., Distante A. (2004). Filter-based feature selection for rail defect detection. Mach. Vis. Appl..

[4] Marino F., Stella E. (2007). ViSyR: A Vision System for Real-Time Infrastructure Inspection.

[5] Jie L., Siwei L., Qingyong L., Hanqing Z., Shengwei R. Real-time rail head surface defect detection: A geometrical approach. Proceedings of the IEEE International Symposium on Industrial Electronics.

[6] Yuan X.C., Wu L.S., Peng Q. (2015). An improved Otsu method using the weighted object variance for defect detection. Appl. Surf. Sci..

[7] Li Q., Ren S. (2012). A real-time visual inspection system for discrete surface defects of rail heads. IEEE Trans. Instrum. Meas..

[8] Feng H., Jiang Z., Xie F., Yang P., Shi J., Chen L. (2014). Automatic fastener classification and defect detection in vision-based railway inspection systems. IEEE Trans. Instrum. Meas..

[9] Papaelias M.P., Lugg M., Roberts C., Davis C. (2009). High-speed inspection of rails using ACFM techniques. NDT E Int..

[10] Clark R. (2004). Rail flaw detection: Overview and needs for future developments. NDT E Int..

[11] Bartoli I., di Scalea F.L., Fateh M., Viola E. (2005). Modeling guided wave propagation with application to the long-range defect detection in railroad tracks. NDT E Int..

[12] Coccia S., Bartoli I., Salamone S., Phillips R., di Scalea F., Fateh M., Carr G. (2009). Noncontact ultrasonic guided wave detection of rail defects. Transp. Res. Rec. J. Transp. Res. Board.

[13] Lanza di Scalea F., Rizzo P., Coccia S., Bartoli I., Fateh M. (2006). Laser-air-coupled hybrid noncontact system for defect detection in rail tracks: Status of FRA prototype development at University of California-San Diego. Transp. Res. Rec..

[14] Alippi C., Casagrande E., Scotti F., Piuri V. (2000). Composite real-time image processing for railways track profile measurement. IEEE Trans. Instrum. Meas..

[15] Babenko P. (2009). Visual Inspection of Railroad Tracks. Ph.D. Thesis.

[16] Deutschl E., Gasser C., Niel A., Werschonig J. Defect detection on rail surfaces by a vision based system. Proceedings of the Intelligent Vehicles Symposium.

[17] Rose J.L., Avioli M.J., Mudge P., Sanderson R. (2004). Guided wave inspection potential of defects in rail. NDT E Int..

[18] Zumpano G., Meo M. (2006). A new damage detection technique based on wave propagation for rails. Int. J. Solids Struct..

[19] Xie X. (2008). A review of recent advances in surface defect detection using texture analysis techniques. Electron. Lett. Comput. Vis. Image Anal..

[20] Forest J., Salvi J. A review of laser scanning three-dimensional digitisers. Proceedings of the IEEE/RSJ International Conference on Intelligent Robots and Systems.

[21] Graebling P., Lallement A., Zhou D.Y., Hirsch E. (2002). Optical high-precision three-dimensional vision-based quality control of manufactured parts by use of synthetic images and knowledge for image-data evaluation and interpretation. Appl. Opt..

[22] Zhang G., He J., Li X. (2005). 3D vision inspection for internal surface based on circle structured light. Sens. Actuators A Phys..

[23] Van Gestel N., Cuypers S., Bleys P., Kruth J.P. (2009). A performance evaluation test for laser line scanners on CMMs. Opt. Lasers Eng..

[24] Wu B., Xue T., Zhang T., Ye S. (2010). A novel method for round steel measurement with a multi-line structured light vision sensor. Meas. Sci. Technol..

[25] Salvi J., Matabosch C., Fofi D., Forest J. (2007). A review of recent range image registration methods with accuracy evaluation. Image Vis. Comput..

[26] Yamany S.M., Farag A.A. Free-form surface registration using surface signatures. Proceedings of the Seventh IEEE International Conference on Computer Vision.

[27] Yamany S.M., Farag A.A. (2002). Surface signatures: An orientation independent free-form surface representation scheme for the purpose of objects registration and matching. IEEE Trans. Pattern Anal. Mach. Intell..

[28] Johnson A.E. (1997). Spin-Images: A Representation for 3-D Surface Matching. Ph.D. Thesis.

[29] Ashbrook A., Fisher R., Werghi N., Robertson C. (1998). Aligning Arbitrary Surfaces Using Pairwise Geometric Histograms. Noblesse Workshop on Non-Linear Model Based Image Analysis.

[30] Zhang D. (1999). Harmonic Shape Images: A 3D Free-Form Surface Representation and Its Applications in Surface Matching. Ph.D. Thesis.

[31] Stein F., Medioni G. (1992). Structural indexing: Efficient 3-D object recognition. IEEE Trans. Pattern Anal. Mach. Intell..

[32] Hummel R.A., Zucker S.W. (1983). On the foundations of relaxation labeling processes. IEEE Trans. Pattern Anal. Mach. Intell..

[33] Li N., Cheng P., Sutton M., McNeill S. (2005). Three-dimensional point cloud registration by matching surface features with relaxation labeling method. Exp. Mech..

[34] Besl P.J., McKay N.D. (1992). Method for registration of 3-D shapes. IEEE Trans. Pattern Anal. Mach. Intell..

[35] Keerthi S.S., Shevade S.K., Bhattacharyya C., Murthy K.R. (2000). A fast iterative nearest point algorithm for support vector machine classifier design. IEEE Trans. Neural Netw..

[36] Rusinkiewicz S., Levoy M. Efficient variants of the ICP algorithm. Proceedings of the Third International Conference on 3-D Digital Imaging and Modeling.

[37] Chetverikov D., Svirko D., Stepanov D., Krsek P. The trimmed iterative closest point algorithm. Proceedings of the 16th International Conference on Pattern Recognition.

[38] Stewart C.V., Tsai C.L., Roysam B. (2003). The dual-bootstrap iterative closest point algorithm with application to retinal image registration. IEEE Trans. Med. Imaging.

[39] Bishop G., Welch G. An introduction to the kalman filter. Proceedings of the Special Interest Group on Computer Graphics and Interactive Techniques.

[40] Julier S.J., Uhlmann J.K. (1997). New extension of the Kalman filter to nonlinear systems. Int. Soc. Opt. Photonics.

[41] Chen Z., Zou H., Jiang H., Zhu Q., Soh Y.C., Xie L. (2015). Fusion of WiFi, smartphone sensors and landmarks using the Kalman filter for indoor localization. Sensors.

[42] Kanungo T., Mount D.M., Netanyahu N.S., Piatko C.D., Silverman R., Wu A.Y. (2002). An efficient k-means clustering algorithm: Analysis and implementation. IEEE Trans. Pattern Anal. Mach. Intell..

[43] Zhang C., Xiao X., Li X., Chen Y.J., Zhen W., Chang J., Zheng C., Liu Z. (2014). White Blood Cell Segmentation by Color-Space-Based K-Means Clustering. Sensors.

[44] Zou Q., Ni L., Wang Q., Hu Z., Li Q., Wang S. (2017). Local Pattern Collocations Using Regional Co-Occurrence Factorization. IEEE Trans. Multimed..

[45] Friedl M.A., Brodley C.E. (1997). Decision tree classification of land cover from remotely sensed data. Remote Sens. Environ..

[46] Pal M., Mather P.M. (2003). An assessment of the effectiveness of decision tree methods for land cover classification. Remote Sens. Environ..

[47] Madzarov G., Gjorgjevikj D., Chorbev I. (2009). A multi-class SVM classifier utilizing binary decision tree. Informatica.

[48] Li Q., Zou Q., Mao Q., Chen X., Li B. (2013). Efficient calibration of a laser dynamic deflectometer. IEEE Trans. Instrum. Meas..

[49] Chang C.C., Lin C.J. (2011). LIBSVM: A library for support vector machines. ACM Trans. Intell. Syst. Technol..

[50] Zou Q., Ni L., Wang Q., Li Q., Wang S. (2017). Robust Gait Recognition by Integrating Inertial and RGBD Sensors. IEEE Trans. Cybern..

